# Examination of Factors Affecting the Likelihood of Whether Individuals Would Purchase Cartilage Conduction Hearing Aids

**DOI:** 10.3390/audiolres13030030

**Published:** 2023-05-11

**Authors:** Shunsuke Takai, Takeshi Sato, Yuya Miyakura, Mika Adachi, Yohei Honkura, Daisuke Yamauchi, Yukio Katori

**Affiliations:** Department of Otolaryngology-Head and Neck Surgery, Tohoku University Graduate School of Medicine, 1-1 Seiryo-machi, Aoba-ku, Sendai 980-8574, Miyagi, Japan

**Keywords:** cartilage conduction hearing aids, air conduction, bone conduction, aided threshold, atresia

## Abstract

Cartilage conduction hearing aids (CC-HAs) are a novel type of hearing aid relying on cartilage conduction, the so-called third auditory conduction pathway. However, CC-HAs have only recently entered routine clinical use, and therefore data on their usefulness are lacking. The purpose of this study was to examine the possibility of assessing whether individual patients would show good adaptation to CC-HAs. Thirty-three subjects (41 ears in total) underwent a free trial of CC-HAs. Age, disease category, and the pure-tone threshold of air and bone conduction, unaided field sound threshold, aided field sound threshold, and functional gain (FG) at 0.25, 0.5, 1, 2, and 4 kHz were compared between patients who subsequently purchased and did not purchase the CC-HAs. Overall, 65.9% of the subjects purchased CC-HAs after the trial. In comparison to non-purchasers, those who decided to purchase CC-HAs showed better pure tone hearing thresholds at high frequencies for both air conduction (2 and 4 kHz) and bone conduction (1, 2, and 4 kHz), as well as for aided thresholds in the sound field (1, 2, and 4 kHz) when using CC-HAs. Therefore, the high-frequency hearing thresholds of subjects trialing CC-HAs might be helpful for identifying those who are likely to benefit from them.

## 1. Introduction

The cartilage conduction pathway was first advocated as a third auditory conduction pathway by Hosoi in 2004 [1]. The cartilage conduction hearing aid (CC-HA) relies on hearing characteristics different from those of conventional air conduction hearing aids, a transducer being placed on the cartilage of the ear to generate sound from the cartilage in the external auditory canal [2,3,4].

In Japan, CC-HAs have been in daily clinical use since November 2017, ahead of any other country in the world [5]. CC-HAs provide adequate hearing amplification without the need for surgery in patients with fibrotic and bony aural atresia, who are unable to wear conventional air conduction hearing aids (AC-HAs). Additionally, CC-HAs avoid local pain and skin irritation caused by high contact pressure because the transducer does not need to be fixed to the patient using a headband, as with conventional bone-conducting hearing aids (BC-HAs) [4]. Therefore, they have been drawing increasing attention as a good alternative for such patients [6,7,8]. However, data about which patients would be most suited for CC-HAs, what the range of hearing that can be sufficiently effective is, and the factors that might influence whether patients would purchase them, are still insufficient [9,10]. 

At our institution, CC-HAs trials and fittings have been available since November 2017. In this study, we evaluated the factors that influenced the decision to purchase CC-HAs, including age, the pure-tone threshold of air and bone conduction before the CC-HA trial, and functional gain. In particular, the effect of bone-conduction hearing threshold on the likelihood of CC-HA purchase has not been assessed previously. The purpose of this study was to investigate the possibility of assessing patient adaptation to CC-HAs based on their demographic characteristics and hearing test results, etc.

## 2. Materials and Methods

This study was approved by the Ethics Committee of Tohoku University Graduate School of Medicine (2022-1-1165), and informed consent was obtained in the form of opt-out on the website. All studies were conducted in accordance with the guidelines of the Declaration of Helsinki (1991). 

**Figure 1:**
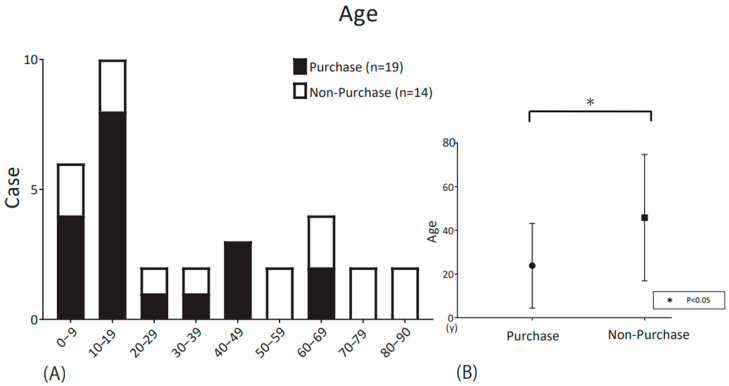
(**A**) Age distribution of the participants according to decade. The number of patients in each decade is shown. The portion of the bar (black) outlined by dashes indicates participants who decided to purchase CC-HAs after the trial period; white indicates non-purchasers. (**B**) Comparison of the ages of CC-HA purchasers and non-purchasers after the trial, represented by the mean and SD.

Thirty-three patients who requested trials of CC-HAs at our institution between November 2017 and July 2022, whose pure tone hearing thresholds with air and bone conduction were testable prior to the CC-HA trial, were included in this study. Of these patients, 20 were male, and 13 were female, with a mean age ± standard deviation (SD) of 33.12 ± 25.55 years (range 4–83 years). Figure 1 shows the distribution of the participants according to age decade.

Audiological thresholds were measured by expert audiometric technicians in a standard sound-attenuated room using a commercially available audiometer (Model AA-H1, RION Co., Ltd., Tokyo, Japan). Pure-tone thresholds were obtained with over-ear headphones to assess AC (125 Hz to 8 kHz) and with a calibrated BC vibrator to assess BC (500 Hz to 4 kHz). Sound-field thresholds (SF) were measured to assess CC-HA-aided and unaided thresholds using warble tones delivered from a loudspeaker located 1 m from the subject at 0° azimuth. For patients with unilateral hearing loss fitted with a CC-HA in only the affected ear, we delivered 70 dB masking noise to the other ear through the headphones to prevent that ear from hearing the test sounds.

An HB-J1CC (Rion Corporation, Kokubunji, Japan) was used for all fittings. Transducers were chosen among ear-chip-embedded, ear-chip-attached, and simple types based on ear condition. The ear tip was made based on an ear mold, allowing for tight attachment to the ear and optimal stability of the transducer. On the other hand, the simple type used double-sided tape for fixation and thus could be applied for any ear condition, regardless of any ear abnormality. 

Subjects were allowed to try their fitted CC-HAs without charge at a follow-up visit two weeks to one month later. At the follow-up visit, they were asked to assess the utility and comfort of the CC-HAs in their daily activities using the speech, spatial, and qualities of hearing scale (SSQ) questionnaires [11] and to undergo measurement of their unaided and aided sound-field thresholds, respectively. Finally, participants were free to choose whether or not to purchase the CC-HA without pressure from the investigator or staff.

The results obtained were compared between purchasers and non-purchasers in terms of age, disease category, SSQ score, the pure-tone threshold of air and bone conduction, the unaided and aided sound field threshold, and functional gain (FG) at 0.25, 0.5, 1, 2, and 4 kHz.

Mann–Whitney U test, chi-square test, and analysis of variance (ANOVA) were employed for statistical analyses using GraphPad Prism 7.0 (GraphPad Prism Software Inc., San Diego, CA, USA). Bonferroni tests were used for post hoc comparisons in ANOVA. The statistical significance level was set at *p* < 0.05.

## 3. Results

This study evaluated the results of the CC-HA trial in 41 ears (33 patients). CC-HAs were purchased for 27 ears (19 patients) and not purchased for 14 ears (14 patients), giving an overall purchase rate of 65.8% for ears that trialed the CC-HAs. Table 1 summarizes the demographic characteristics of the study participants and their hearing assessments before and after listening to the CC-HAs. There were no significant differences between groups with regard to sex and functional gains. Significant differences were found for age, clinical characteristics, and unaided and aided sound field thresholds.

Figure 1 shows the age distribution of purchasers and non-purchasers by ten-year age group. Among the subjects, 19 were purchasers, and 14 were non-purchasers. The mean age ± standard deviation (SD) of the purchasers was 23.8 ± 18.9 years (range 4–67 years), whereas that of the non-purchasers was 45.8 ± 27.9 years (range 4–83 years) (Table 1). The purchasers were significantly younger than the non-purchasers (*p* < 0.05; Mann–Whitney U-test).

**Figure 2:**
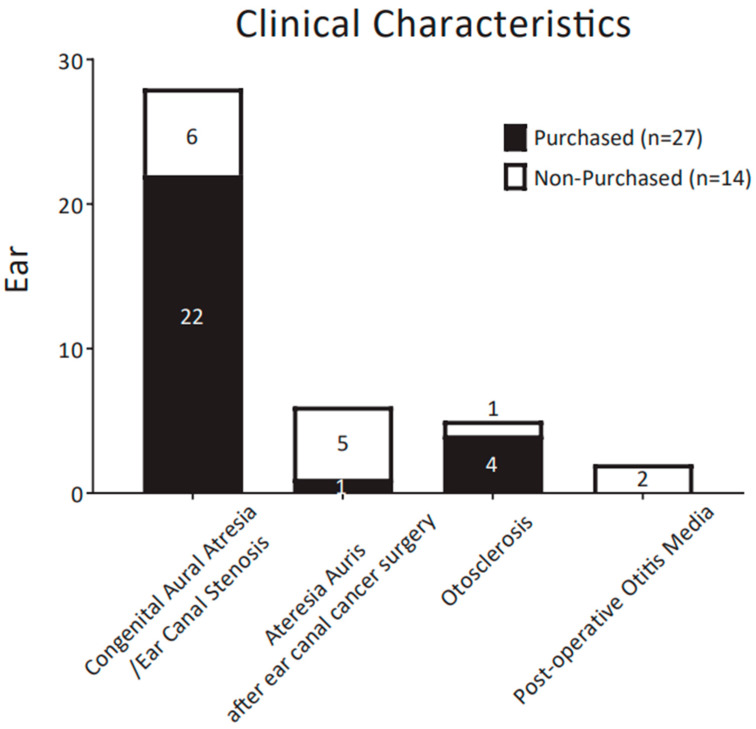
Distribution of the participants’ clinical characteristics. The number of patients in each decade is shown. The portion of the bar (black) outlined by dashes indicates participants who decided to purchase CC-HAs after the trial period; white indicates non-purchasers.

Figure 2 compares the clinical characteristics of purchasers and non-purchasers in the form of a histogram. Congenital aural atresia/ear canal stenosis was present in 28 ears (28/41, 68.3%). The next most frequent conditions were atresia auris after ear canal cancer surgery (6/41, 14.6%), otosclerosis (5/41, 12.2%), and postoperative otitis media (2/41, 4.9%). The purchase rate was 79% (22/28 ears) in the congenital aural atresia/ear canal stenosis group and 17% (1/6 ears) in the acquired atresia auris group. In the otosclerosis group and postoperative otitis media group, the purchase rate was 80% (4/5) and 0% (0/2 ears), respectively. Congenital atresia/ear canal stenosis had a significantly higher purchase rate among study participants compared to the other conditions (Table 1).

**Figure 3:**
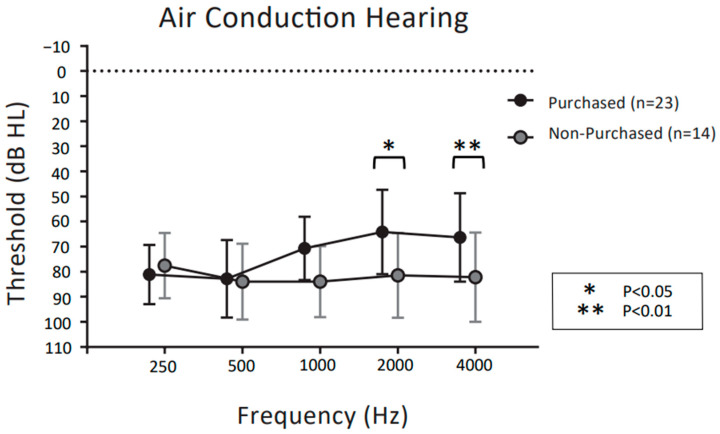
Comparison of air conduction hearing in pure tone auditory between CC-HA purchasers and non-purchasers, represented by the mean and SD. The dotted polygonal line (black) indicates participants who decided to purchase CC-HAs after the trial period; grey indicates non-purchasers.

**Figure 4:**
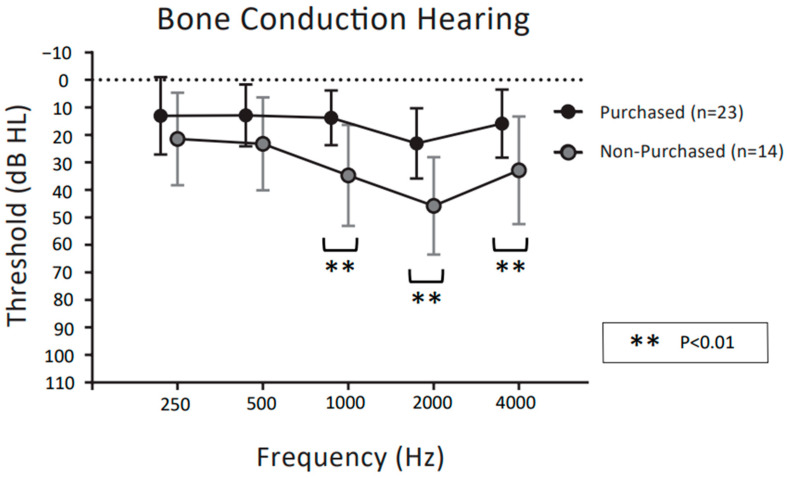
Comparison of bone conduction hearing in pure tone auditory between CC-HA purchasers and non-purchasers, represented by the mean and SD. The dotted polygonal line (black) indicates participants who decided to purchase CC-HAs after the trial period; grey indicates non-purchasers.

Figure 3 and Figure 4 show the mean pure tone audiometry values for air and bone conduction prior to the CC-HAs trial, respectively, at 0.25, 0.5, 1, 2, and 4 kHz in the purchasers and non-purchasers. Two preschool children (4 ears) could not be tested. Therefore, 31 patients (37 ears) were evaluated.

For the air conduction thresholds in Figure 3, two-way ANOVA demonstrated significant main effects for frequency (F (4,175) = 2.687, *p* = 0.033) and purchase rate (F (1,175) = 14.66, *p* < 0.001). The interaction between frequency and purchase rate was significant (F (4,175) = 3.366, *p* < 0.05). Post hoc comparisons showed that purchasers had significantly better air conduction thresholds than non-purchasers at 2 kHz and 4 kHz (*p* < 0.05 post hoc Bonferroni test). For the bone conduction thresholds in Figure 4, two-way ANOVA demonstrated significant main effects for frequency (F (4,175) = 7.823, *p* < 0.001) and purchase rate (F (1,175) = 52.24, *p* < 0.001). The interaction between frequency and purchase rate was not significant (F (4,175) = 1.651, *p* = 0.164). Post hoc comparisons showed that purchasers had significantly better bone conduction thresholds than non-purchasers at 1 kHz, 2 kHz, and 4 kHz (*p* < 0.01 post hoc Bonferroni test).

**Figure 5:**
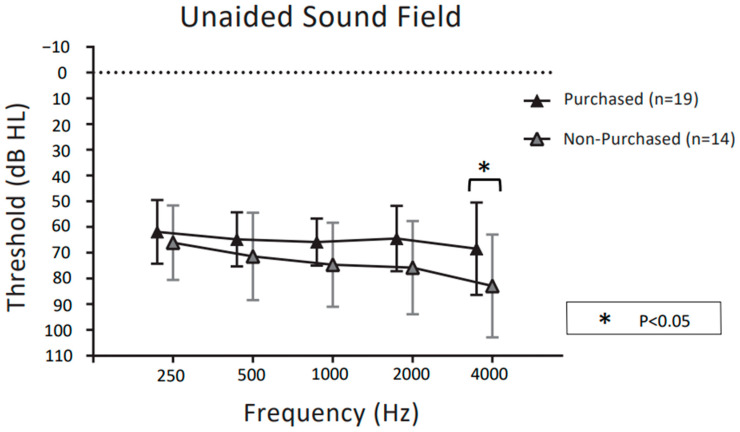
Comparison of unaided sound field thresholds between CC-HA purchasers and non-purchasers, represented by the mean and SD. The dotted polygonal line (black) indicates participants who decided to purchase CC-HAs after the trial period; grey indicates non-purchasers.

**Figure 6:**
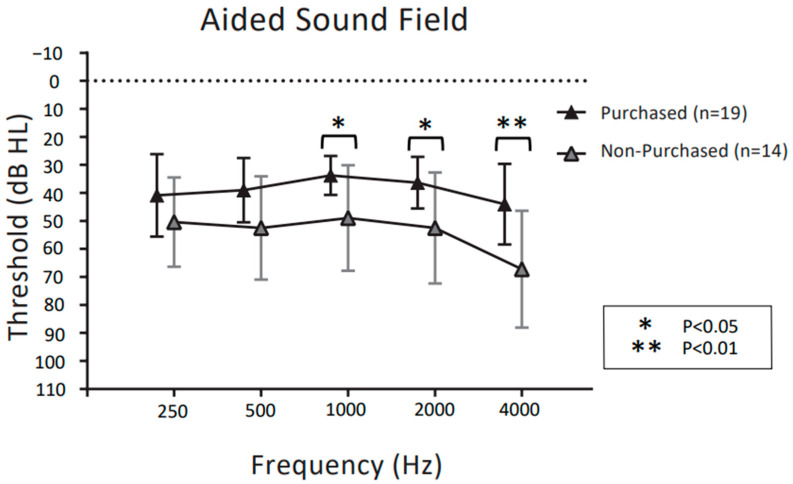
Comparison of aided sound field thresholds between CC-HA purchasers and non-purchasers, represented by the mean and SD. The dotted polygonal line (black) indicates participants who decided to purchase CC-HAs after the trial period; grey indicates non-purchasers.

Figure 5 and Figure 6 show unaided and aided thresholds in the sound field at the follow-up visit for purchasers and non-purchasers of CC-HAs, respectively. For the unaided thresholds in the sound field in Figure 5, two-way ANOVA demonstrated significant main effects for frequency (F (4,155) = 2.612, *p* = 0.038) and purchase rate (F (1,155) = 15.15, *p* < 0.001). There was no significant interaction between frequency and purchase rate (F (4,155) = 0.574, *p* = 0.68).

Post hoc comparisons showed that purchasers had significantly better unaided thresholds in the sound fields than non-purchasers only at 4 kHz (*p* < 0.05 post hoc Bonferroni test). For the aided thresholds in the sound field in Figure 6, two-way ANOVA demonstrated significant main effects for frequency (F (4,155) = 4.068, *p* = 0.0036) and purchase rate (F (1,155) = 42.90, *p* < 0.001). The interaction between frequency and purchase rate was not significant (F (4,155) = 0.876, *p* = 0.480). Post hoc comparisons showed that purchasers had significantly better bone conduction thresholds than non-purchasers at 1 kHz, 2 kHz, and 4 kHz (*p* < 0.05 post hoc Bonferroni test).

**Figure 7:**
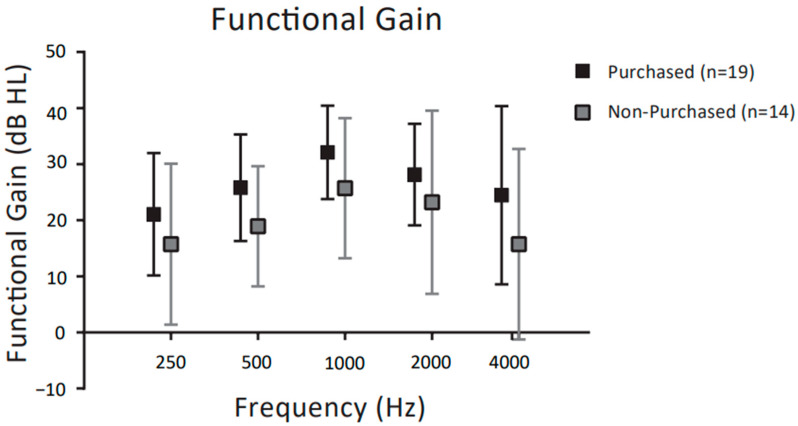
Comparisons of the functional gain achieved using CC-HAs between purchasers and non-purchasers, represented by the mean and SD. The dotted (black) outline indicates participants who purchased CC-HAs after the trial period; grey indicates non-purchasers.

Figure 7 shows the functional gain (FG) for purchasers and non-purchasers of CC-HAs, respectively. Two-way ANOVA revealed significant main effects for frequency (F (4,155) = 4.068, *p* = 0.036) and purchase rate (F (1,155) = 42.90, *p* < 0.001), but no significant interaction between frequency and purchase rate (F (4,155) = 0.876, *p* = 0.48). Further post hoc comparisons showed that purchasers did not have significantly higher FG than non-purchasers at any frequency (*p* < 0.05 post hoc Bonferroni test).

**Figure 8:**
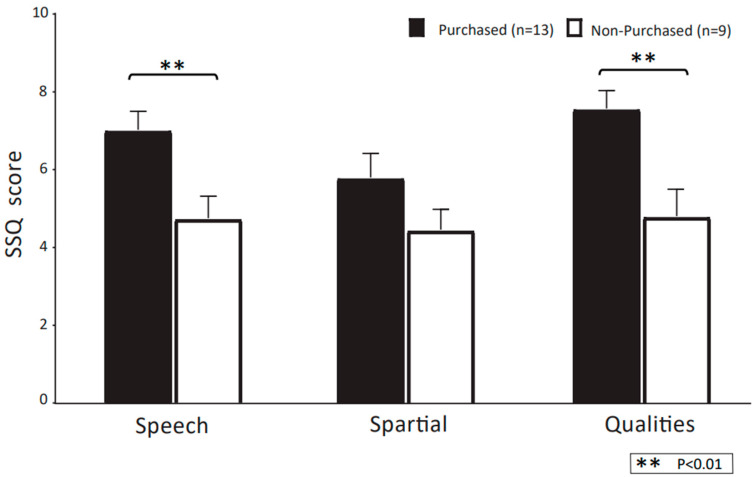
Comparisons of the speech, spatial, and qualities of hearing scale (SSQ) using CC-HAs between purchasers and non-purchasers, represented by the mean and SEM. The dotted (black) outline indicates participants who purchased CC-HAs after the trial period; white indicates non-purchasers.

Figure 8 shows the mean scores of each SSQ questionnaire for CC-HA purchasers and non-purchasers. Purchasers had significantly higher mean scores for SSQ speech and SSQ quality than non-purchasers (*p* < 0.05; Mann–Whitney U-test). On the other hand, SSQ spatial did not differ significantly between purchasers and non-purchasers.

## 4. Discussion

This study aimed to evaluate the efficacy of CC-HAs for hearing-impaired patients who were unable to use conventional air- or bone-conduction hearing aids, and the factors that contribute to the decision to purchase them. In particular, we were interested in whether test results obtained prior to the start of CC-HA use, such as those of pre-trial pure tone audiometry, could be used to assess whether patients would adapt well to CC-HAs.

The overall rate of CC-HA purchase in this study was 65.9% (27/41 ears), which is within the ranges reported previously [6,7,9,10]. In the congenital aural atresia/ear canal stenosis group, the purchase rate was 79% (22/28 ears), a significantly higher purchase rate than for other conditions (Table 1). Nishimura reported high CC-HA purchase rates of 86% and 78% in the Bi-Closed and Uni-Closed groups, respectively [9]. This was similar to the rate in our congenital aural atresia/ear canal stenosis group. On the other hand, in the group with acquired atresia auris after ear canal cancer surgery, the purchase rate was lower at 17% (1/6 ears). In that group, the average air conduction threshold was poor, with a hearing loss of more than 70 dB in almost all cases. Nishiyama et al. also reported that the rate of CC-HA purchase in patients with canal stenosis, including both congenital and acquired atresia, was lower in individuals with severe hearing loss exceeding 70 dB [6]. In summary, it is suggested that CC-HAs may not be sufficiently effective for the improvement of hearing in individuals with severe hearing loss of 70 dB or more.

In the present study, CC-HA purchasers were significantly younger than non-purchasers. Regarding the relationship between purchase rate and age, it has been reported that purchasers are significantly younger than non-purchasers among patients with hearing loss due to unilateral atresia auris [9,10]. This result might be due to the more perceived benefits of binaural hearing for communication and education in younger than in older individuals [12,13]. Furthermore, in Japan, social support for children with mild/moderate hearing loss is often provided for the purchase of hearing aids. This may account for the difference in purchase rates between younger and older children.

In this study, more than 80% of the subjects trialing CC-HAs had aural atresia/ear canal stenosis. In previous studies, CC-HAs have been used most frequently in patients with congenital atresia or acquired atresia due to surgical treatment, such as ear canal cancer, and have been reported to improve hearing [3,4,14,15,16]. In addition, CC-HAs were trialed in five ears with otosclerosis, and the subsequent purchase rate was 80% (4/5 ears). Although CC-HAs may be a good option for otosclerosis patients, there have been few reports of trials for such patients [6], and further investigations are required.

With regard to pure tone hearing thresholds, these were significantly better in purchasers than in non-purchasers at frequencies of 2 kHz and 4 kHz for air conduction and at 1 kHz, 2 kHz, and 4 kHz for bone conduction. This means that for both air conduction and bone conduction, the pure tone hearing thresholds at higher frequencies were significantly better in the individuals who purchased CC-HAs than in those who did not. Previous reports have often compared CC-HAs unaided and aided with sound field thresholds [4,6,7,9,10], and thus the results suggest that residual thresholds for high tone frequencies may be an important and novel factor affecting the likelihood of CC-HA purchase.

For aided thresholds in the sound field, these were significantly better among CC-HA purchasers than among non-purchasers at 1 kHz, 2 kHz, and 4 kHz. Previous reports suggested that purchasers had significantly better sound field assistance thresholds than non-purchasers at lower frequencies of 0.25 kHz and 0.5 kHz, in contrast to the present results. However, one study comparing the transmission efficiency of cartilage conduction (CC), air conduction (AC), and bone conduction (BC) revealed that the threshold increases were significantly better for BC than for CC at frequencies of 1 kHz and 2 kHz [14]. Therefore, CC has a lower transmission efficiency than BC at higher frequencies, which may support our present results. Certainly, CC-HAs may provide less effective hearing compensation than BC-HAs. However, CC-HAs are small and lightweight, and there is no pain or occurrence of skin laceration due to transducer pressure with a fixation headband, which is common with BC-HAs [3,4,17,18]. This feature is considered one of the advantages of CC-HAs.

No significant difference in FG was found between purchasers and non-purchasers at any frequency, suggesting that threshold increases in the two groups were similar. Previous reports have also indicated that CC-HAs improved hearing thresholds at all frequencies, regardless of the purchase outcome of CC-HA trials [6,7,17]. In summary, this trial of CC-HAs for patients with hearing loss demonstrated a similar functional gain in both non-purchasers and purchasers. However, the functional gain may have been insufficient for hearing impairment at higher frequencies because of the lower transmission efficiency attributable to the transmission features of the CC. In the previous study [19] comparing hearing test results between CC-HAs and BC-HAs, BC-HAs had significantly better functional gains at high-frequency ≥ 1 KHz. The results of this previous study supported our findings. Therefore, the high-frequency hearing thresholds of subjects undergoing CC-HA trials might be a helpful criterion for identifying individuals for whom CC-HAs would be effective.

In the present study, we evaluated hearing aid use using the SSQ questionnaire to assess the usefulness and comfort of the CC-HAs. Purchasers had significantly higher SSQ speech and SSQ quality than non-purchasers. On the other hand, SSQ spatial was not significantly different between purchasers and non-purchasers, but there was a tendency for advantages among purchasers. Although the evaluation of CC-HAs using questionnaires has been studied in the past using “Evaluation of hearing before and after wearing a hearing aid, [4]” there are still few reports on this topic, and further studies are needed.

The limitations of this study included its small sample size, lack of speech audiometry assessment, and absence of any comparison between CC-HAs and other hearing aids (e.g., AC-HAs and BC-HAs). It has already been reported that speech audiometry assessments are improved, as well as the hearing threshold [4,17]. The economic background of patients, which may also influence whether they purchase CC-HAs, was also not examined. We suggest that residual hearing in the high-frequency range is a potentially useful criterion for indicating individuals who would benefit from CC-HAs. However, for further confirmation, future studies with a larger number of cases are needed.

## 5. Conclusions

This study investigated the factors influencing the decision of patients to purchase CC-HAs on the basis of trials performed in our department. Overall, 62.2% of the subjects purchased CC-HAs after the trials. Purchasers had better air-conduction and bone-conduction thresholds for pure tone hearing thresholds than non-purchasers at high frequencies, as well as for aided thresholds in the sound field when fitted with CC-HAs. Hearing-impaired patients with better pure tone hearing thresholds at relatively high frequencies may be better candidates for CC-HAs. However, there are still few reports investigating the clinical adaptation of CC-HAs. Further comparisons with other types of hearing aids, such as AC-HAs and BC-HAs, are needed.

## Figures and Tables

**Table 1:** Demographic characteristics of participants according to group: purchase or non-purchase.

Characteristics	Purchase Case (n = 19)	Non Purchase Case (n = 14)	*p* Value
Sex, male; female	11; 8	9; 5	0.710 ^†^
Age at fitting (yr, Mean ± SD)	23.8 ± 18.9	45.8 ± 27.9	0.037 ^‡^
Clinical characteristics Congenital canal atresia/stenosis; others	17; 2	9; 5	0.004 ^†^
Average unaided sound field thresholds * (dB HL, Mean ± SD)	65.9 ± 11.2	76.2 ± 14.8	0.036 ^‡^
Average Aided sound field thresholds * (dB HL, Mean ± SD)	38.2 ± 9.2	55.3 ± 16.3	<0.001 ^‡^
Average Functional Gain * (dB HL, Mean ± SD)	27.6 ± 9.2	20.9 ± 11.6	0.063 ^‡^

* Average of hearing thresholds at 500, 1000, 2000, and 4000 Hz; † Chi-square test; and ‡ Mann–Whitney U test.

## Data Availability

Not applicable.
